# Surfing the Hyperbola Equations of the Steady-State Farquhar–von Caemmerer–Berry C_3_ Leaf Photosynthesis Model: What Can a Theoretical Analysis of Their Oblique Asymptotes and Transition Points Tell Us?

**DOI:** 10.1007/s11538-019-00676-z

**Published:** 2019-12-23

**Authors:** Jon Miranda-Apodaca, Emilio L. Marcos-Barbero, Rosa Morcuende, Juan B. Arellano

**Affiliations:** Departamento de Estrés Abiótico, Instituto de Recursos Naturales y Agrobiología de Salamanca (IRNASA-CSIC), Cordel de Merinas, 40-52, 37008 Salamanca, Spain

**Keywords:** FvCB model, Mesophyll conductance, Leaf photosynthesis, Net CO_2_ assimilation rate, Resistance to CO_2_ diffusion, Rubisco

## Abstract

The asymptotes and transition points of the net CO_2_ assimilation (*A*/*C*_i_) rate curves of the steady-state Farquhar–von Caemmerer–Berry (FvCB) model for leaf photosynthesis of C_3_ plants are examined in a theoretical study, which begins from the exploration of the standard equations of hyperbolae after rotating the coordinate system. The analysis of the *A*/*C*_i_ quadratic equations of the three limitation states of the FvCB model—abbreviated as *A*_c_, *A*_j_ and *A*_p_—allows us to conclude that their oblique asymptotes have a common slope that depends only on the mesophyll conductance to CO_2_ diffusion (*g*_m_). The limiting values for the transition points between any two states of the three limitation states c, j and p do not depend on *g*_m_, and the results are therefore valid for rectangular and non-rectangular hyperbola equations of the FvCB model. The analysis of the variation of the slopes of the asymptotes with *g*_m_ casts doubts about the fulfilment of the steady-state conditions, particularly, when the net CO_2_ assimilation rate is inhibited at high CO_2_ concentrations. The application of the theoretical analysis to extended steady-state FvCB models, where the hyperbola equations of *A*_c_, *A*_j_ and *A*_p_ are modified to accommodate nitrogen assimilation and amino acids export via the photorespiratory pathway, is also discussed.

**Electronic supplementary material:**

The online version of this article (10.1007/s11538-019-00676-z) contains supplementary material, which is available to authorized users.

## Introduction

The steady-state Farquhar–von Caemmerer–Berry (FvCB) leaf photosynthesis model is broadly recognised by plant biologists and physiologists as one of the most useful models to assess in vivo the net CO_2_ assimilation rate (*A*) of plant leaves as a function of CO_2_ concentration (*C*) under different environmental cues. The initial FvCB model was first described in the 1980s for C_3_ plants (Farquhar et al. 1980), then modified to include the triose phosphate utilisation (Sharkey 1985a, b) and later extended to other works on C_4_ plants, antisense transgenic plants, the effect of bicarbonate pumps at the chloroplast envelope and global climate change, among others (Bellasio et al. 2016; Price et al. 2011; von Caemmerer 2000; Wullschleger 1993). Together with the basic rectangular hyperbolic FvCB model (Farquhar et al. 1980; Sharkey 1985a, b), other non-rectangular hyperbolic, exponential and empirical steady-state models have also been described (Duursma 2015; Ethier and Livingston 2004; Goudriaan 1979; von Caemmerer 2000). In the basic FvCB model, the steady-state CO_2_ assimilation rate proceeds at the minimum of three limitation rates denoted as *A*_c_, *A*_j_ and *A*_p_, which depend on the activity of the ribulose-1,5-bisphosphate carboxylase/oxygenase (Rubisco), the ribulose-1,5-bisphosphate regeneration and the triose phosphate utilisation, hereafter abbreviated as states c, j and p.

In the basic FvCB model, the analysis of the net CO_2_ assimilation rate did not consider the (apparent) mesophyll conductance to CO_2_ diffusion (*g*_m_)—hereafter defined as the conductance for CO_2_ diffusion from the intercellular space to the site of Rubisco carboxylation assuming that photorespiratory and respiratory CO_2_ release occurs in the same compartment as Rubisco carboxylation (von Caemmerer 2013)—and thus, its value was assumed to be infinite. Consequently, the CO_2_ concentration in the intercellular (or substomatal) space (*C*_i_) was set equal to the CO_2_ concentration at the site of Rubisco carboxylation (*C*_c_) and both rate curves, *A*/*C*_c_ and *A*/*C*_i_, were not distinguished between each other. The inclusion of a finite value for *g*_m_ into the initial FvCB model transforms *A*_c_ and *A*_j_ into quadratic equations. This transformation was indeed demonstrated to provide a more accurate estimation of the values for the maximum carboxylation rate (*V*_cmax_) of Rubisco and the maximum electron transport *J*_max_ under steady-state conditions (Ethier and Livingston 2004; Niinemets et al. 2009; von Caemmerer 2000). From a mathematical point of view, the main difference between the equations of the *A*/*C*_c_ and *A*/*C*_i_ rate curves is that the latter are non-rectangular hyperbolae, whose curvature shape in the first quadrant of the Cartesian coordinate system depends on the magnitude of *g*_m_.

Nowadays, *A*/*C*_i_, instead of *A*/*C*_c_, rate curves are extensively used in the estimation of biochemical parameters from leaf photosynthesis, where *g*_m_ is assumed to be finite and purely diffusional and not to depend on the CO_2_ concentration inside the leaf. However, the assumption that *g*_m_ remains constant has been challenged in some studies and new extensions have been incorporated into the FvCB model (Flexas et al. 2007; Tholen et al. 2012). For instance, *g*_m_ was proposed to depend on the ratio of mitochondrial CO_2_ release to chloroplast CO_2_ uptake and to decrease particularly at low *C*_i_ (Tholen et al. 2012), although other factors, such as the intracellular arrangements of chloroplasts and mitochondria in C_3_ leaves, were later included in a more generalised model to better explain the dependence of *g*_m_ on the above ratio (Yin and Struik 2017). A decrease in the values for *g*_m_ was also observed in response to an increase in *C*_i_ (Flexas et al. 2007). In this latter study, either modifications in chloroplast shape, which could prevent the chloroplast association with the cell surface, or the involvement of aquaporins, which could facilitate CO_2_ diffusion across cell membranes by a pH-dependent process, was proposed to regulate the variation of *g*_m_. Besides, *g*_m_ is tightly co-regulated with the stomatal conductance (*g*_s_) (Flexas et al. 2008). *g*_s_ varies with both the atmospheric CO_2_ concentration and the limitation state (Buckley 2017). The value of *g*_s_ declines with increased atmospheric CO_2_ concentration under RuBP regeneration-limited photosynthesis, but, in contrast, it increases with increased atmospheric CO_2_ concentration under Rubisco-limited photosynthesis (Medlyn et al. 2011).

When the *A*/*C*_i_ rate curves of the FvCB model are analysed under steady-state conditions and the photorespiratory and respiratory CO_2_ release is also assumed to take place at the site of the Rubisco carboxylation, the quadratic equations for *A*_c_, *A*_j_ and *A*_p_ (see “Appendix 1”, Eqs. A8–A10) can be fitted following different approaches, where *g*_m_ is taken as a constant parameter (Duursma 2015; Gu et al. 2010; Sharkey 2016; Su et al. 2009). Some of the nonlinear fitting methods require starting from initial guessed parameters and letting the fit improve with successive iterations, while others constrain the *C*_i_ values at which the transition point between c and j occurs. A wealth of data on the transition point between the states c and j indicates that its value is species- and season-dependent, and so it should not be constrained in the fitting method (Duursma 2015; Miao et al. 2009; Zeng et al. 2010). The above fitting methods also take up that the *A*/*C*_i_ rate curves reach asymptotic values for *A* at supraoptimal CO_2_ concentration, when there is experimental evidence for the inhibition of the net CO_2_ assimilation rate by CO_2_ itself at high concentrations (Woo and Wong 1983). Also, some of these fitting methods make use of approximate estimations for *J*_max_ and *T*_p_—where *T*_p_ stands for the rate of phosphate release in triose phosphate utilisation—when *C*_c_ approaches infinity in an *A*/*C*_c_ rate curve (Su et al. 2009) or they reasonably assume that the order of the three limitation states along the *C*_i_ axis is the same as along the *C*_c_ axis (Gu et al. 2010). Dynamic models of photosynthesis are also suitable to analyse the leaf CO_2_ assimilation response under fluctuating environmental stimuli such as sunlight irradiance, atmospheric CO_2_ concentration or stomatal response to light (Bellasio 2019; Morales et al. 2018; Noe and Giersch 2004); however, they add complexity to the analysis or they have not been developed completely to date.

The simplicity of the quadratic equations for *A*_c_, *A*_j_ and *A*_p_ still makes the steady-state FvCB model very useful in fitting approaches to estimate biochemical parameters from leaf photosynthesis (Duursma 2015; Gu et al. 2010; Sharkey 2016; Su et al. 2009). After nearly 40 years of research on the FvCB model, its quadratic equations still hide mathematical features of interest to establish when this model becomes short or when an extended FvCB model would be more suitable for the estimation of the biochemical parameters. On the mathematical analysis of the FvCB model we present here, the rotation of the coordinate system has been a key strategy to reach the conclusion that the quadratic equations of the FvCB model cannot explain the inhibition of the net CO_2_ assimilation rate at very high *C*_i_. Also, the mathematical analysis of the limiting conditions for the transition points between *A*_c_, *A*_j_ and *A*_p_ shows that they do not depend on the finite value of *g*_m_.

## Computer Analysis

The computer algebra system Wolfram Mathematica v. 10.3 (Wolfram Research 2015) was used to program scripts to solve analytically the asymptotes and transition points of *A*/*C*_c_ and *A*/*C*_i_ rate curves of the three limitation states c, j and p. Comparative analyses were performed with hyperbolae in standard form after the rotation of the coordinates. The scripts were also run to plot representative *A*/*C*_c_ and *A*/*C*_i_ rate curves. The chosen and finite values for the kinetic constants for Rubisco and other biochemical parameters in the simulations are in the range of those experimentally determined for different C_3_ plant species (Jahan et al. 2014; von Caemmerer 2000). A list of definitions is given in Table 1 for the sake of clarity.

**Table 1:** List of biochemical parameters used with their definition and units

Symbol	Definition	Units
*A*	Net CO_2_ assimilation rate	µmol m^−2^ s^−1^
*A*_c_	Net CO_2_ assimilation rate assuming Rubisco limitation	µmol m^−2^ s^−1^
*A*_j_	Net CO_2_ assimilation rate assuming ribulose-1,5-bisphosphate regeneration limitation	µmol m^−2^ s^−1^
*A*_p_	Net CO_2_ assimilation rate assuming triose phosphate use limitation	µmol m^−2^ s^−1^
$A_{*1}^{\text{xy}}$	*A* at the transition point between any two limitation states (*x* and *y*) of the three states c, j and p in the fourth (or third) quadrant of the Cartesian coordinate system. The symbol * stands for chloroplast space (c), intercellular (i) or atmosphere (a)	µmol m^−2^ s^−1^
$A_{*2}^{\text{xy}}$	As $A_{*1}^{\text{xy}}$, except that the transition point takes place in the first quadrant of the Cartesian coordinate system.	µmol m^−2^ s^−1^
*C*_c_	Chloroplast CO_2_ concentration	Pa
*C*_i_	Intercellular CO_2_ concentration	Pa
*C*_a_	Atmospheric CO_2_ concentration	Pa
$C_{*1}^{\text{xy}}$	CO_2_ concentration at the transition point between any two limitation states (*x* and *y*) of the three states c, j and p in the fourth (or third) quadrant of the Cartesian coordinate system. * stands for chloroplast (c), intercellular space (i) or atmosphere (a)	Pa
$C_{*2}^{\text{xy}}$	As $C_{*1}^{\text{xy}}$, except the transition point takes place in the first quadrant of the Cartesian coordinate system	Pa
$C_{{{\text{c2}} \to 1}}^{\text{xy}}$	Value of the CO_2_ concentration when $C_{\text{c2}}^{\text{xy}}$ approaches $C_{\text{c1}}^{\text{xy}}$	Pa
$C_{{{\text{c2}} \to \infty }}^{\text{xy}}$	Value of the CO_2_ concentration when $C_{\text{c2}}^{\text{xy}}$ approaches infinity	Pa
$C_{{{\text{i2}} \to 1}}^{\text{xy}}$	Value of the CO_2_ concentration when $C_{\text{i2}}^{\text{xy}}$ approaches $C_{\text{i1}}^{\text{xy}}$	Pa
$C_{{{\text{i2}} \to \infty }}^{\text{xy}}$	Value of the CO_2_ concentration when $C_{\text{i2}}^{\text{xy}}$ approaches infinity	Pa
*J*	Electron transport rate	µmol m^−2^ s^−1^
*J*_max_	Maximal electron transport rate	µmol m^−2^ s^−1^
*K*_c_	Rubisco Michaelis–Menten constant for carboxylation	Pa
*K*_o_	Rubisco Michaelis–Menten constant for oxygenation	Pa
*K*_co_	Apparent Michaelis–Menten constant	Pa
*O*	Oxygen concentration	Pa
*R*_d_	Respiration rate in the light	µmol m^−2^ s^−1^
*r*_m_	Apparent mesophyll resistance to CO_2_ diffusion (see the Introduction section). Inverse of *g*_m_	Pa µmol^−1^ m^2^ s
*r*_s_	Stomatal resistance to CO_2_ diffusion. Inverse of *g*_s_	Pa µmol^−1^ m^2^ s
*T*_p_	Triose phosphate export rate from chloroplasts	µmol m^−2^ s^−1^
*V*_cmax_	Maximum carboxylation rate	µmol m^−2^ s^−1^
$y_{\text{asyn}}^{\text{x}}$	Asymptote with horizontal slope of *A*_x_, where *x* stands for c, j or p	µmol m^−2^ s^−1^Pa^−1^
$y_{\text{asyp}}^{\text{x}}$	Asymptote with positive slope of *A*_x_, where *x* stands for c, j or p	µmol m^−2^ s^−1^Pa^−1^
*α*	Fraction of glycerate that does not return to chloroplasts through the photorespiratory cycle	Dimensionless
*β*	Angle of rotation of the coordinate system	Dimensionless
Γ*	Chloroplast CO_2_ photocompensation point	Pa

## Results and Discussion

### Brief Description of the Asymptotes and Transition Points of the Rectangular Hyperbola Equations of the Basic FvCB Model

According to the basic FvCB model for leaf photosynthesis in C_3_ plants (Farquhar et al. 1980; Sharkey 1985a, b), the hyperbola equations of the dependence of the net CO_2_ assimilation rate on the CO_2_ concentration at the site of the Rubisco carboxylation (i.e. *A*/*C*_c_) are as follows:

1 $$ A = V_{\text{c}} \left( {1 - 0.5\phi } \right) - R_{\text{d}} = \hbox{min} \left\{ {A_{\text{c}} ,A_{\text{j}} ,A_{\text{p}} } \right\} $$

with

2 $$ A_{\text{c}} = \frac{{V_{\text{cmax}} (C_{\text{c}} - \varGamma^{*} )}}{{C_{\text{c}} + K_{\text{co}} }} - R_{\text{d}} , $$

3 $$ A_{\text{j}} = \frac{{J(C_{\text{c}} - \varGamma^{*} )}}{{4C_{\text{c}} + 8\varGamma^{*} }} - R_{\text{d}} , $$

4 $$ A_{\text{p}} = \frac{{3T_{\text{p}} (C_{\text{c}} - \varGamma^{*} )}}{{C_{\text{c}} - (1 + 3\alpha )\varGamma^{*} }} - R_{\text{d}} , $$

where *A* proceeds at a minimum of the three limitation rates *A*_c_, *A*_j_ and *A*_p_. The equations of the three rate curves in the basic FvCB model are branches of rectangular hyperbolae opening upwards and downwards or left and right, where the coordinate system has been rotated 45° (Appendix 1). The two asymptotes of each of the hyperbolic equations (Eqs. 2–4) are perpendicular to each other with slopes 0 and infinite (Table 2). An elemental analysis of the transition points between the rate equations of the three limitation states gives the following sets of solutions:

**Table 2:** Summary of the values and equations of the centre, asymptotes and bisecting lines describing the rectangular and non-rectangular hyperbolae of the FvCB model for C_3_ plants

	Vertical asymptote	Horizontal asymptote	Centre	Bisecting line
*Rectangular hyperbolae*				
*A*_c_	$C_{\text{i}}^{\text{c}} = - K_{\text{co}}$	$A_{\text{asyn}}^{\text{c}} = V_{\text{cmax}} - R_{\text{d}}$	$\left( { - K_{\text{co}} , \, V_{\text{cmax}} - R_{\text{d}} } \right)$	$A_{\text{bis}}^{\text{c}} = - \left( {C_{\text{i}} + K_{\text{co}} } \right) + V_{\text{cmax}} - R_{\text{d}}$
*A*_j_	$C_{\text{i}}^{\text{j}} = - 2\varGamma^{*}$	$A_{asyn}^{j} = {J \mathord{\left/ {\vphantom {J 4}} \right. \kern-0pt} 4} - R_{\text{d}}$	$\left( { - 2\varGamma^{*} , \, {J \mathord{\left/ {\vphantom {J 4}} \right. \kern-0pt} 4} - R_{\text{d}} } \right)$	$A_{\text{bis}}^{\text{j}} = - \left( {C_{\text{i}} + 2\varGamma^{*} } \right) + {J \mathord{\left/ {\vphantom {J 4}} \right. \kern-0pt} 4} - R_{\text{d}}$
*A*_p_	$C_{\text{i}}^{\text{p}} = \left( {1 + 3\alpha } \right)\varGamma^{*}$	$A_{\text{asyn}}^{\text{p}} = 3T_{\text{p}} - R_{\text{d}}$	$\left( {\left( {1 + 3\alpha } \right)\varGamma^{*} , \, 3T_{\text{p}} - R_{\text{d}} } \right)$	$A_{\text{bis}}^{\text{p}} = \left( {C_{\text{i}} - \left( {1 + 3\alpha } \right)\varGamma^{*} } \right) + 3T_{\text{p}} - R_{\text{d}}$

	Oblique asymptote	Horizontal asymptote	Centre	Bisecting line
*Non-rectangular hyperbolae*				
*A*_c_	$A_{\text{asyp}}^{\text{c}} = \frac{{C_{\text{i}} + K_{\text{co}} }}{{r_{\text{m}} }}$	$A_{\text{asyn}}^{\text{c}} = V_{\text{cmax}} - R_{\text{d}}$	$\left( {\left( {V_{\text{cmax}} - R_{\text{d}} } \right)r_{\text{m}} - K_{\text{co}} , \, V_{\text{cmax}} - R_{\text{d}} } \right)$	$A_{\text{bis}}^{\text{c}} = - \left( {r_{\text{m}} + \sqrt {1 + r_{\text{m}}^{2} } } \right)( {C_{\text{i}} - ( {V_{\text{cmax}} - R_{\text{d}} } )r_{\text{m}} + K_{\text{co}} } ) + V_{\text{cmax}} - R_{\text{d}}$
*A*_j_	$A_{\text{asyp}}^{\text{j}} = \frac{{C_{\text{i}} + 2\varGamma^{*} }}{{r_{\text{m}} }}$	${{A_{\text{asyn}}^{\text{j}} = J} {/ {\vphantom {{A_{\text{asyn}}^{\text{j}} = J} 4}} \kern-0pt} 4} - R_{\text{d}}$	$\left( {\left( {{J \mathord{\left/ {\vphantom {J 4}} \right. \kern-0pt} 4} - R_{\text{d}} } \right)r_{\text{m}} - 2\varGamma^{*} , \, {J \mathord{\left/ {\vphantom {J 4}} \right. \kern-0pt} 4} - R_{\text{d}} } \right)$	$A_{\text{bis}}^{\text{j}} = - \left( {r_{\text{m}} + \sqrt {1 + r_{\text{m}}^{2} } } \right)\left( {C_{\text{i}} - \left( {{J \mathord{\left/ {\vphantom {J 4}} \right. \kern-0pt} 4} - R_{\text{d}} } \right)r_{\text{m}} + 2\varGamma^{*} } \right) + {J \mathord{\left/ {\vphantom {J 4}} \right. \kern-0pt} 4} - R_{\text{d}}$
*A*_p_	$A_{\text{asyp}}^{\text{p}} = \frac{{C_{\text{i}} - \left( {1 + 3\alpha } \right)\varGamma^{*} }}{{r_{\text{m}} }}$	$A_{\text{asyn}}^{\text{p}} = 3T_{\text{p}} - R_{\text{d}}$	$\left( {\left( {3T_{\text{p}} - R_{\text{d}} } \right)r_{\text{m}} + \left( {1 + 3\alpha } \right)\varGamma^{*} , \, 3T_{\text{p}} - R_{\text{d}} } \right)$	$A_{\text{bis}}^{\text{p}} = \left( { - r_{\text{m}} + \sqrt {1 + r_{\text{m}}^{2} } } \right)\left( {C_{\text{i}} - \left( {3T_{\text{p}} - R_{\text{d}} } \right)r_{\text{m}} - \left( {1 + 3\alpha } \right)\varGamma^{*} } \right) + 3T_{\text{p}} - R_{\text{d}}$

For $$ A_{\text{c}} = A_{\text{j}} $$,

5a, 5b $$ C_{\text{c1}}^{\text{cj}} = \varGamma^{*} \quad {\text{and}}\quad A_{\text{c1}}^{\text{cj}} = - R_{\text{d}} , $$

6a, 6b $$ C_{\text{c2}}^{\text{cj}} = \frac{{8V_{\text{cmax}} \varGamma^{*} - JK_{\text{co}} }}{{J - 4V_{\text{cmax}} }}\quad {\text{and}}\quad A_{\text{c2}}^{\text{cj}} = \frac{{(J - 4R_{\text{d}} )K_{\text{co}} + (J + 8R_{\text{d}} - 12V_{\text{cmax}} )\varGamma^{*} }}{{4K_{\text{co}} - 8\varGamma^{*} }} $$

for $$ A_{\text{j}} = A_{\text{p}} $$,

7a, 7b $$ C_{\text{c1}}^{\text{jp}} = \varGamma^{*} \quad {\text{and}}\quad A_{\text{c1}}^{\text{jp}} = - R_{\text{d}} , $$

8a, 8b $$ C_{\text{c2}}^{\text{jp}} = \frac{{(J + 24T_{\text{p}} + 3\alpha J)\varGamma^{*} }}{{J - 12T_{\text{p}} }}\quad {\text{and}}\quad A_{\text{c2}}^{\text{jp}} = \frac{{12T_{\text{p}} + \alpha J - 4R_{\text{d}} (1 + \alpha )}}{4(1 + \alpha )}, $$

and for $$ A_{\text{c}} = A_{\text{p}} $$,

9a, 9b $$ C_{\text{c1}}^{\text{cp}} = \varGamma^{*} \quad {\text{and}}\quad A_{\text{c1}}^{\text{cp}} = - R_{\text{d}} , $$

10a $$ C_{\text{c2}}^{\text{cp}} = \frac{{(1 + \alpha )V_{\text{cmax}} \varGamma^{*} + 3T_{\text{p}} K_{\text{co}} }}{{V_{\text{cmax}} - 3T_{\text{p}} }}\quad {\text{and}} $$

10b $$ A_{\text{c2}}^{\text{cp}} = \frac{{(3(T_{\text{p}} + \alpha V_{\text{cmax}} ) - (1 + \alpha )R_{\text{d}} )\varGamma^{*} + (3T_{\text{p}} - R_{\text{d}} )K_{\text{co}} }}{{K_{\text{co}} + (1 + \alpha )\varGamma^{*} }} $$

Together with the transition points $(C_{{{\text{c}}2}}^{xy} ,A_{{{\text{c}}2}}^{xy} )$ between any two limitation states of the three states c, j and p (superscripts *x* and *y*) in the first quadrant of the Cartesian coordinate system (subscript 2), there is a common transition point $(C_{{{\text{c}}1}}^{xy} ,A_{{{\text{c}}1}}^{xy} )$ in the fourth quadrant (subscript 1) when $\alpha \ne 0$ ($0 \le \alpha \le 1$). Carbon and electron requirements for the assimilation of nitrogen and export of amino acids through the photorespiratory pathway (Busch et al. 2018) are not addressed here, and the standard definition for *α* in the basic FvCB model remains (see below for further discussion).

### Dependence of the Oblique Asymptotes of the Non-rectangular Hyperbola (or Quadratic) Equations of the FvCB Model on *r*_m_

The mathematical analysis becomes more challenging if *A*/*C*_i_, instead of *A*/*C*_c_, rate curves are used. When steady-state conditions for CO_2_ diffusion are achieved, *A*_c_, *A*_j_ and *A*_p_ can be determined after the substitution of *C*_c_ for *C*_i_ using the equation $A = {{(C_{\text{i}} - C_{\text{c}} )} \mathord{\left/ {\vphantom {{(C_{\text{i}} - C_{\text{c}} )} {r_{\text{m}} }}} \right. \kern-0pt} {r_{\text{m}} }}$ according to Fick’s diffusion law, where the finite and “constant” mesophyll resistance to CO_2_ diffusion is $r_{\text{m}} = {1 \mathord{\left/ {\vphantom {1 {g_{\text{m}} }}} \right. \kern-0pt} {g_{\text{m}} }}$. Quadratic equations are obtained for *A*_c_, *A*_j_ and *A*_p_ (Eqs. A8–A10). They are now non-rectangular hyperbolae opening upwards and downwards, for the case of *A*_c_ and *A*_j_, and left and right, for the case of *A*_p_, where the coordinate system has now been rotated anticlockwise an angle, here denoted *β* (Appendix 1). One of the two asymptotes from each non-rectangular hyperbola is parallel to the horizontal axis, but the other is now oblique with a slope exactly equal to the mesophyll conductance to CO_2_ diffusion, i.e. $g_{\text{m}} = {1 \mathord{\left/ {\vphantom {1 {r_{\text{m}} }}} \right. \kern-0pt} {r_{\text{m}} }}$, a result which is valid for *A*_c_ (von Caemmerer 2000) and also for *A*_j_ and *A*_p_. This conclusion is reached following the analysis of the coefficients of the quadratic equations obtained after the anticlockwise rotation of the coordinate system by *β*. When Eqs. A6 and A7 are compared with Eqs. A8–A10, some key features emerge: firstly, the summation of the coefficients of $C_{\text{i}}^{2}$ is equal to zero and, secondly, the second coefficient of the quadratic equations is, in fact, the summation of the two asymptotes of each hyperbola (Appendix 1). The equations of the two asymptotes for *A*_c_, *A*_j_ and *A*_p_ are therefore summarised as follows:

11a, 11b $$ y_{\text{asyp}}^{\text{c}} = \frac{{C_{\text{i}} + K_{\text{co}} }}{{r_{\text{m}} }}\quad {\text{and}}\quad y_{\text{asyn}}^{\text{c}} = V_{\text{cmax}} - R_{\text{d}} , $$

12a, 12b $$ y_{\text{asyp}}^{\text{j}} = \frac{{C_{\text{i}} + 2\varGamma^{*} }}{{r_{\text{m}} }} \quad {\text{and}}\quad y_{\text{asyn}}^{\text{j}} = {J \mathord{\left/ {\vphantom {J 4}} \right. \kern-0pt} 4} - R_{\text{d}} ,\;{\text{and}} $$

13a, 13b $$ y_{\text{asyp}}^{\text{p}} = \frac{{C_{\text{i}} - \left( {1 + 3\alpha } \right)\varGamma^{*} }}{{r_{\text{m}} }}\quad {\text{and}}\quad y_{\text{asyn}}^{\text{p}} = 3T_{\text{p}} - R_{\text{d}} , $$

where $y_{\text{asyp}}^{x}$ and $y_{\text{asyn}}^{x}$ stand for the oblique and horizontal asymptotes of *A*_c_, *A*_j_ and *A*_p_, respectively.

**Fig. 1:**
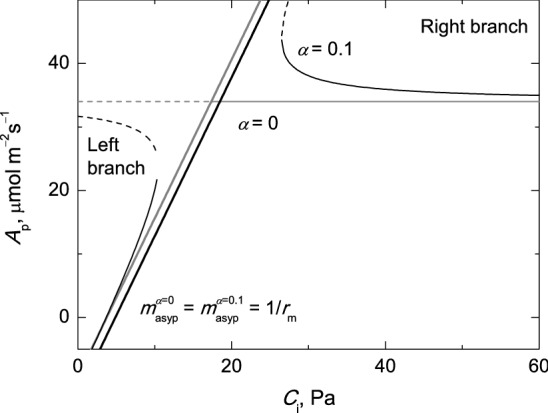
Representative *A*_p_ rate curves for the non-rectangular FvCB model for C_3_ plants with two different values for *α* and their corresponding oblique asymptotes. The negative root (thin solid line) and the positive root (thin dashed line) of the quadratic equation of *A*_p_ together with its oblique asymptote (thick solid line) are in grey for $\alpha = 0$. The negative root (thin solid line) and the positive root (thin dashed line) of the quadratic equation of *A*_p_ together with its oblique asymptote (thick solid line) are in black for $\alpha = 0.1$. The simulation was performed using the following values for the biochemical parameters: *T*_p_, 12 μmol m^−2^ s^−1^; *R*_d_, 2 μmol m^−2^ s^−1^; *r*_m_, 0.4 Pa µmol^−1^ m^2^ s; Γ^*^, 3.74 Pa. Note: The negative root of the branch opening left and the positive root of the branch opening right of *A*_p_ for $\alpha = 0$ are overlaid with its oblique asymptote

It is worth noting that the use of $\alpha = 0$ directly in Eq. 4 is an oversimplification of *A*_p_. The oblique asymptote of *A*_p_ is present, even when *α* is assumed to be equal to zero (Eq. 13a). The intersection between the two asymptotes of *A*_p_ (i.e. $y_{\text{asyp}}^{\text{p}}$ and $y_{\text{asyn}}^{\text{p}}$), in particular when $\alpha = 0$, gives a limiting value below which *C*_i_ is meaningless. In fact, the approximation $A_{\text{p}} = 3T - R_{\text{d}}$ is not valid in the whole *C*_i_ domain between $\varGamma^{*} \le C_{\text{i}} \le \infty$. The discontinuity is more obvious when $\alpha \ne 0$ because there is a *C*_i_ domain for which no real values for *A*_p_ can be obtained. The suitable *C*_i_ domain for the nonlinear fitting of *A*_p_ in the FvCB model is thus confined to the negative root of its branch opening right (Fig. 1), a result which is also in line with the study by Gu et al. (2010). When $\alpha \ne 0$, the values of the negative root of the *A*_p_ branch opening right decrease as *C*_i_ increases.

### The Limiting Conditions for the Transition Points in the FvCB Model Do Not Depend on *r*_m_

The transition points for the negative roots of the quadratic equations for *A*/*C*_i_ (Eqs. A8–A10) can be solved mathematically and written in a simple form making use of the analytical solutions (Eqs. 5–10) for *A*/*C*_c_ as follows:

For $$ A_{\text{c}} = A_{\text{j}} $$,

14a, 14b $$ C_{{{\text{i}}1}}^{\text{cj}} = C_{{{\text{c}}1}}^{\text{cj}} + r_{\text{m}} A_{\text{c1}}^{\text{cj}} \quad {\text{and}}\quad A_{\text{i1}}^{\text{cj}} = A_{\text{c1}}^{\text{cj}} , $$

15a, 15b $$ C_{\text{i2}}^{\text{cj}} = C_{\text{c2}}^{\text{cj}} + r_{\text{m}} A_{\text{c2}}^{\text{cj}} \quad {\text{and}}\quad A_{\text{i2}}^{\text{cj}} = A_{\text{c2}}^{\text{cj}} $$

for $$ A_{\text{j}} = A_{\text{p}} $$,

16a, 16b $$ C_{\text{i1}}^{\text{jp}} = C_{\text{c1}}^{\text{jp}} + r_{\text{m}} A_{\text{c1}}^{\text{jp}} \quad {\text{and}}\quad A_{\text{i1}}^{\text{jp}} = A_{\text{c1}}^{\text{jp}} $$

17a, 17b $$ C_{\text{i2}}^{\text{jp}} = C_{\text{c2}}^{\text{jp}} + r_{\text{m}} A_{\text{c2}}^{\text{jp}} \quad {\text{and}}\quad A_{\text{i2}}^{\text{jp}} = A_{\text{c2}}^{\text{jp}} $$

and for $$ A_{\text{c}} = A_{\text{p}} $$,

18a, 18b $$ C_{\text{i1}}^{\text{cp}} = C_{\text{c1}}^{\text{cp}} + r_{\text{m}} A_{\text{c1}}^{\text{cp}}  \quad {\text{and}}\quad A_{\text{i1}}^{\text{cp}} = A_{\text{c1}}^{\text{cp}} $$

19a, 19b $$ C_{\text{i2}}^{\text{cp}} = C_{\text{c2}}^{\text{cp}} + r_{\text{m}} A_{\text{c2}}^{\text{cp}} \quad {\text{and}}\quad A_{\text{i2}}^{\text{cp}} = A_{\text{c2}}^{\text{cp}} $$

**Fig. 2:**
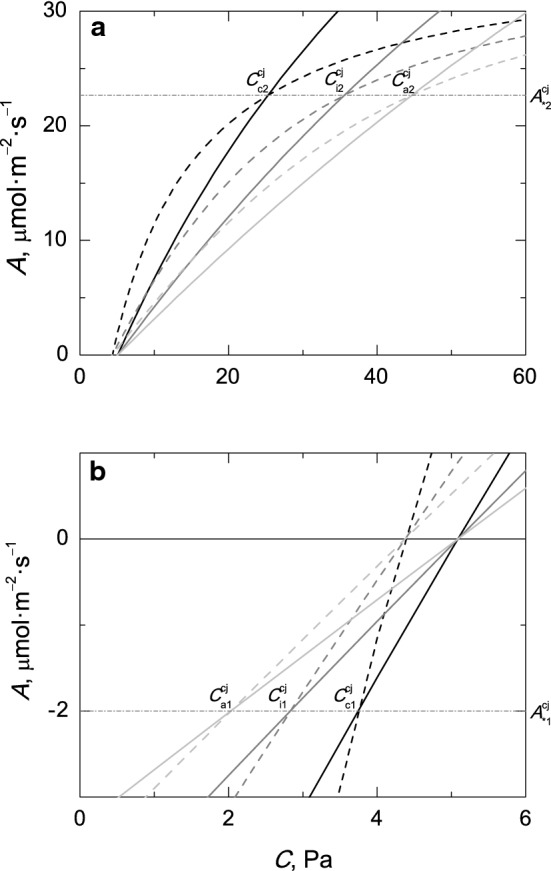
Transition points $\left( {C_{ * 2}^{\text{cj}} ,A_{ * 2}^{\text{cj}} } \right)$ and $\left( {C_{ * 1}^{\text{cj}} ,A_{ * 1}^{\text{cj}} } \right)$ between *A*_c_ (solid lines) and *A*_j_ (dashed lines) for the rectangular and non-rectangular hyperbolic equations of the FvCB model for C_3_ plants in the first (**a**) and fourth (**b**) quadrants of the Cartesian coordinate system. In the transition points, the obtained values for the net CO_2_ assimilation rate ($A_{ * 1}^{\text{cj}}$ and $A_{ * 2}^{\text{cj}}$) remain constant (dotted-dashed lines), while the values for the CO_2_ concentration ($C_{ * 1}^{\text{cj}}$ and $C_{ * 2}^{\text{cj}}$) at the transition points depend on the type of *A*/*C* rate curve (*A*/*C*_c_, black lines; *A*/*C*_i_, grey lines; and *A*/*C*_a_, light grey lines). The asterisks stand for the CO_2_ concentration at the carboxylation site (**c**), intercellular space (i) or the atmosphere (**a**). The simulation was performed using the following values for the biochemical parameters: *V*_cmax_, 100 μmol m^−2^ s^−1^; *J*, 150 μmol m^−2^ s^−1^; *R*_d_, 2 μmol m^−2^ s^−1^; *r*_m_, 0.45 Pa µmol^−1^ m^2^ s; *r*_**s**_, 0.4 Pa µmol^−1^ m^2^ s; *K*_co_, 62.1 Pa; Γ^*^, 3.74 Pa

The above solutions could be further extended to include the stomatal resistance to CO_2_ diffusion ($r_{\text{s}} = {1 \mathord{\left/ {\vphantom {1 {g_{\text{s}} }}} \right. \kern-0pt} {g_{\text{s}} }}$) in *A*_c_, *A*_j_ and *A*_p_ (see Appendix 2). Figure 2 summarises the changes in the transition points in the first and fourth quadrants of the Cartesian coordinate system when the resistance(s) to CO_2_ diffusion is included in the net CO_2_ assimilation rate curves. For the sake of clarity, only *A*_c_ and *A*_j_ are shown. The analytical values for the assimilation rates ($A_{*1}^{xy}$ and $A_{*2}^{xy}$) at the transition points remain constant, while the values for the CO_2_ concentration increase in the first quadrant (and decrease in the fourth quadrant) when the resistance(s) to CO_2_ diffusion increases. The solution $(\varGamma^{*} , - R_{\text{d}} )$ for the transition point $(C_{{{\text{c}}1}}^{xy} ,A_{{{\text{c}}1}}^{xy} )$ is restricted to the fourth quadrant of the Cartesian coordinate system when the rectangular hyperbolae (Eqs. 2–4) of the basic FvCB model are used. However, it should not be surprising to find out this analytical transition point $(C_{{{\text{i}}1}}^{xy} ,A_{{{\text{i}}1}}^{xy} )$ in the third quadrant under conditions for which $R_{\text{d}} r_{\text{m}} > \varGamma^{*}$ when dealing with the quadratic equations of the FvCB model.

In the mathematical analysis, it can be observed that the common transition point $(C_{{{\text{i}}1}}^{xy} ,A_{{{\text{i}}1}}^{xy} )$ between the three states c, j and p is always present; in contrast, the transition points $(C_{{{\text{i}}2}}^{xy} ,A_{{{\text{i}}2}}^{xy} )$ depend on the biochemical parameters and might not be present in the net CO_2_ assimilation rate curves. Two limiting conditions can now be investigated in order to analyse all the possible combinations between the transition points between *A*_c_, *A*_j_ and *A*_p_ regardless of the value of *r*_m_. One is that $C_{\text{i2}}^{xy}$ approaches $C_{\text{i1}}^{xy}$ (i.e. $C_{{{\text{i2}} \to 1}}^{xy}$) and another is that $C_{\text{i2}}^{xy}$ approaches infinity (i.e. $C_{{{\text{i2}} \to \infty }}^{xy}$). The equation $C_{\text{i1}}^{xy} = C_{\text{i2}}^{xy}$ has to be solved for the analysis of the first limiting condition, whereas only the values for the denominator of the first summand (i.e. $C_{\text{c2}}^{xy}$) of $C_{{{\text{i}}2}}^{xy}$ (Eqs. 15a–19a) have to be inspected to analyse the second limiting condition. In the analysis, the constraint $K_{\text{co}} > 2\varGamma^{*}$ is imposed based on the values reported for C_3_ plants (von Caemmerer 2000); consequently, there are no finite values for the biochemical parameters of the second summand (i.e. $r_{\text{m}} A_{\text{c2}}^{xy}$) of $C_{{{\text{i}}2}}^{xy}$ (Eqs. 15a–19a) that can make this summand approach infinity.

The ratios between the biochemical parameters to reach the above limiting conditions for the rectangular equations of an *A*/*C*_c_ rate curve (i.e. $C_{{{\text{c2}} \to 1}}^{xy}$ and $C_{{{\text{c2}} \to \infty }}^{xy}$) can be derived straightforward from Eqs. 5a–10a. The results are as follows:

For $C_{{{\text{c2}} \to 1}}^{\text{cj}}$ and $C_{{{\text{c2}} \to \infty }}^{\text{cj}}$, respectively,

20a, 20b $$ V_{\text{cmax}} = \frac{{J\left( {K_{\text{co}} + \varGamma^{*} } \right)}}{{12\varGamma^{*} }}\quad {\text{and}}\quad V_{\text{cmax}} = \frac{J}{4} $$

for $C_{{{\text{c2}} \to 1}}^{\text{jp}}$ and $C_{{{\text{c2}} \to \infty }}^{\text{jp}}$, respectively,

21a, 21b $$ J = - \frac{{12T_{\text{p}} }}{\alpha }\quad {\text{and}}\quad J = 12T_{\text{p}} $$

and for $C_{{{\text{c2}} \to 1}}^{\text{cp}}$ and $C_{{{\text{c2}} \to \infty }}^{\text{cp}}$, respectively,

22a, 22b $$ V_{\text{cmax}} = - \frac{{T_{\text{p}} \left( {K_{\text{co}} + \varGamma^{*} } \right)}}{{\alpha \varGamma^{*} }}\quad {\text{and}}\quad V_{\text{cmax}} = 3T_{\text{p}} $$

Among the above ratios (Eqs. 20a–22b), the ratios between the biochemical parameters for $C_{{{\text{c2}} \to 1}}^{\text{jp}}$ and $C_{{{\text{c2}} \to 1}}^{\text{cp}}$ are of non-biochemical significance. They imply that there should be conditions for which one could expect triose phosphate import to chloroplasts (i.e. *T*_p_ < 0). In fact, if these transition points are analysed, particularly, in a non-rectangular *A*/*C*_i_ rate curve, one can observe that both transitions, $(C_{\text{i1}}^{\text{cp}} ,A_{\text{i1}}^{\text{cp}} )$ and $(C_{\text{i1}}^{\text{jp}} ,A_{\text{i1}}^{\text{jp}} )$, occur with the negative root of the hyperbolic branch opening left of *A*_p_, for which the *C*_i_ domain is not applicable as indicated above. (Further details are given in Fig. S1 of Online Resource.)

When the rest of the ratios between the biochemical parameters are now investigated in the non-rectangular hyperbola equations of the FvCB model—where $r_{\text{m}}$ is finite, $0 < r_{\text{m}} < \infty$—two solutions are in fact found for any equation like $C_{{{\text{i}}2}}^{xy} = C_{{{\text{i}}1}}^{xy}$, of which only one also fulfils the condition $A_{{{\text{i}}2}}^{xy} = A_{{{\text{i}}1}}^{xy}$ (data not shown). The analysis indeed shows that the correct ratios between the biochemical parameters for $C_{{{\text{i}}2 \to 1}}^{xy}$ and $C_{{{\text{i}}2 \to \infty }}^{xy}$ are the same as those found for $C_{{{\text{c}}2 \to 1}}^{xy}$ and $C_{{{\text{c}}2 \to \infty }}^{xy}$ (Eqs. 20a–22b). This means that the ratios between the biochemical parameters in the two limiting conditions do not depend on the value of *r*_m_. The limiting conditions for the transition points can therefore be reduced as follows:

23 $$ V_{\text{cmax}} = \frac{{J\left( {K_{\text{co}} + \varGamma^{*} } \right)}}{{12\varGamma^{*} }}, $$

24 $$ J = 4V_{\text{cmax}} ,\quad {\text{and}} $$

25 $$ J = 12T_{\text{p}} $$

**Fig. 3:**
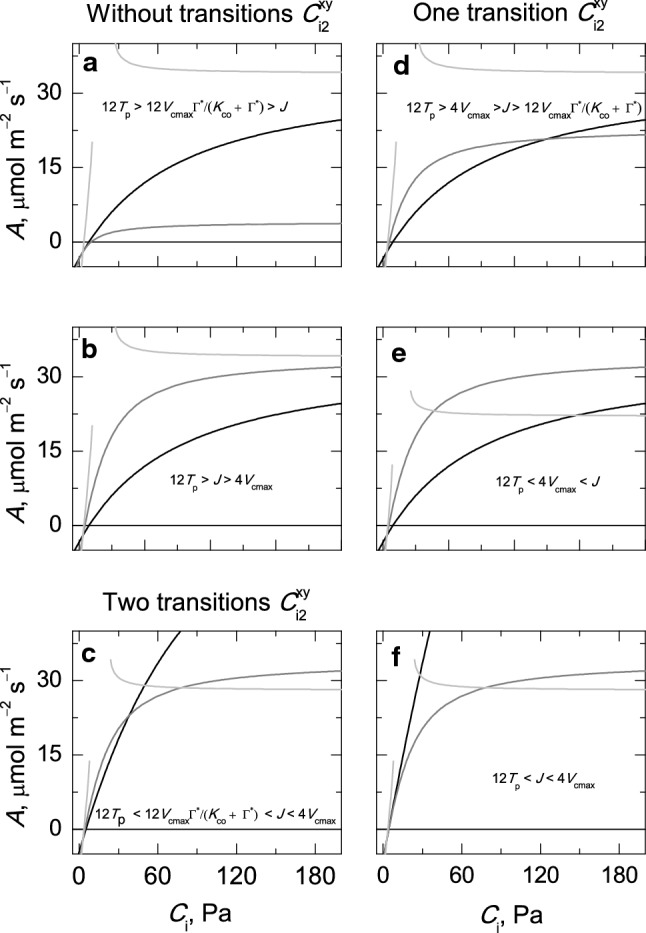
Ratios between the limiting values of the biochemical parameters of a representative *A*/*C*_i_ rate curve, where the summation of resistances to CO_2_ diffusion is included (i.e. $0 < r_{\text{m}} < \infty$), for which there are no transition points between the three states c, j and p (**a**, **b**) and two transition points (**c**) or there is only one transition point (**d**–**f**) in the first quadrant of the Cartesian coordinate system $(C_{\text{i2}}^{\text{xy}} ,A_{\text{i2}}^{\text{xy}} )$. The symbols x and y stand for any of the three limitation states c, j and p. The simulation was performed using the following values for the biochemical parameters: *V*_cmax_, 36–100 μmol m^−2^ s^−1^; *J*, 24–144 μmol m^−2^ s^−1^; *T*_p_, 8–12 μmol m^−2^ s^−1^; *R*_d_, 2 μmol m^−2^ s^−1^; *r*_m_, 0.4 Pa µmol^−1^ m^2^ s; *K*_co_, 62.1 Pa; Γ^*^, 3.74 Pa

The graphic representation of *A*_c_, *A*_j_ and *A*_p_ for *A*/*C*_i_ rate curves shows, firstly, that there are no experimental ratios for the biochemical parameter for which *A*_p_ (with $T_{\text{p}} > 0$) can be the only limitation state along the domain $\varGamma^{*} - r_{\text{m}} R_{\text{d}} < C_{\text{i}} < \infty$ and, secondly, there are ratios between the biochemical parameters for which *A*_c_ or *A*_j_ can be the only limitation state along the domain $\varGamma^{*} - r_{\text{m}} R_{\text{d}} < C_{\text{i}} < \infty$ (Fig. 3a, b). Additional ratios between the biochemical parameters can be found for which there are one or two transition points in the first quadrant of Cartesian coordinate system (Fig. 3c–f). The latter ratios are equivalent to those discussed before for *A*/*C*_c_ rate curves (Gu et al. 2010). Regardless of the number of transition points (0, 1 or 2) that the ratios of the biochemical parameters can yield between the three limitation states in the first quadrant of the Cartesian coordinate system, the transition points in the fourth (or third) quadrant are always present.

### Analysis of the Inhibition of the Net CO_2_ Assimilation Rate at High CO_2_ Concentrations

If the steady-state FvCB model is strictly followed, one can state, first, that the slopes of the oblique asymptotes of the non-rectangular hyperbolae only depend on $g_{\text{m}} = {1 \mathord{\left/ {\vphantom {1 {r_{\text{m}} }}} \right. \kern-0pt} {r_{\text{m}} }}$, while the slopes of the horizontal asymptotes of *A*_c_, *A*_j_ and *A*_p_ remain unchanged regardless of the value for *g*_m_ (Eqs. 11a–13b) and, second, the slopes of the bisecting lines (Table 2) of *A*_c_, *A*_j_ and *A*_p_ correspond with the angle (or the perpendicular angle) of the rotation of the coordinate system that makes the summation of the coefficients of $C_{\text{i}}^{2}$ equal to zero (Eqs. A11 and A12). This means that there are *no* mathematical solutions for the quadratic equations of *A*_c_, *A*_j_ and *A*_p_ (Eqs. A8–A10) in the FvCB model for which the slopes of the horizontal asymptotes can be modified to reach negative values. The fraction of glycerate ($\alpha \ne 0$) that does not return to chloroplasts through the photorespiratory cycle (Harley and Sharkey 1991) makes *A*_p_ decrease as *C*_i_ increases, but the slope of the horizontal asymptote of *A*_p_ remains unchanged, no matter what value *α* ($0 \le \alpha \le 1$) has. This indicates that *A*_p_ must finally reach a constant value as *C*_i_ increases. This conclusion also applies to the extended FvCB model described in the study by Busch et al. (2018), where the parameter *α* of the basic FvCB model is replaced with two new parameters α_G_ and α_S_ that stand for the proportion of glycolate carbon taken out of the photorespiratory pathway as glycine, and the proportion taken out as serine, respectively. Although α_G_ and α_S_ might not be constant and depend on the photorespiratory pathway and the reduction of supplied nitrate (Busch et al. 2018), the new equations for the three limitation states remain, from a mathematical point of view, as rectangular hyperbolae with horizontal asymptotes equivalent to those summarised in Table 2. Likewise, the extension of the FvCB model using $r_{\text{m}}$ as a flux-weighted quantity that depends on mitochondrial respiration and photorespiration effects does not explain the inhibition of *A*/*C*_i_ at high *C*_i_ either (Tholen et al. 2012).

Despite what has been said above, there are lines of experimental evidence that indicate that negative slopes can be indeed observed in *A*/*C*_i_ rate curves. Woo and Wong (1983) showed that supraoptimal CO_2_ concentrations inhibited the net CO_2_ assimilation in cotton plants, and they proposed that an acidification mechanism mediated by CO_2_ could affect both the thylakoid electron transport and the activity of key enzymes of the Calvin–Benson–Bassham cycle (Kaiser and Heber 1983; Ögren and Evans 1993). At this point, one could speculate on some mathematical explanations for negative slopes in experimental *A*/*C*_i_ rate curves. In the first place, one could wonder whether other rotation of the coordinates—different from the one that yields Eqs. A28 and A29—would be possible under steady-state conditions, which rendered negative asymptotes instead of horizontal asymptotes. If this were possible, the summation of the coefficients of $C_{\text{i}}^{2}$ (Eqs. A11 and A12) would not be zero and so the chosen fitting method should start from extended quadratic equations as Eqs. A6 and A7, where at least four parameters defining the hyperbola equation and an angle of rotation had to be determined. In this case, the angle of rotation should depend on *g*_m_ together with other biochemical parameters. Alternatively, one could wonder whether the steady-state conditions do not hold along the whole *C*_i_ domain, particularly at supraoptimal CO_2_ concentrations. If this were the case, one can assert that the equation $A = {{(C_{\text{i}} - C_{\text{c}} )} \mathord{\left/ {\vphantom {{(C_{\text{i}} - C_{\text{c}} )} {r_{\text{m}} }}} \right. \kern-0pt} {r_{\text{m}} }}$ is not always valid. So, *A* decreases at supraoptimal CO_2_ concentrations because either *r*_m_ is not only diffusional and so it increases as *C*_i_ increases (Flexas et al. 2007) or the photosynthetic activity is indeed inhibited by CO_2_ acidification (Kaiser and Heber 1983; Ögren and Evans 1993; Woo and Wong 1983). Reliable nonlinear fittings of *A*_c_ and *A*_j_ of the FvCB model can thus be possibly obtained under steady-state conditions using standard approaches (Duursma 2015; Gu et al. 2010; Sharkey 2016; Su et al. 2009); however, the use supraoptimal CO_2_ concentrations to fit *A*_p_ might cast doubts on the fitted biochemical parameters if evidence for negative slopes in the experimental *A*/*C*_i_ rate curves is observed. Based on the variation of *g*_m_ with *C*_i_ (Flexas et al. 2007), other nonlinear fitting approaches proposed the combination of gas exchange methods with chlorophyll fluorescence-based methods to estimate *g*_m_ by using only data within the j state (Yin and Struik 2009).

## Conclusions

The analysis of the steady-state FvCB model for C_3_ plants starting from the standard equations of hyperbolae after rotating the coordinate system has disclosed some features hidden in the quadratic equations of *A*_c_, *A*_j_ and *A*_p_ of the *A*/*C*_i_ rate curves. In particular, academic interest has been the angle of the rotation of the coordinate system from which it has been established that the oblique asymptotes of the three limitation rate curves share a common slope whose value depends only on *g*_m_. *A*_p_ always has an oblique asymptote regardless of the value of *α*. The limiting conditions for the transition points in the FvCB model do not depend on *g*_m_. The hyperbola equations of *A*_c_, *A*_j_ and *A*_p_ in the FvCB model or in some of the extended steady-state FvCB models here discussed can only provide horizontal asymptotes when the CO_2_ concentration approaches infinity when, in contrast, there is experimental evidence for negative slopes in *A*/*C*_i_ rate curves at high CO_2_ concentrations. This leads us to the conclusion that extended quadratic equations containing a $C_{\text{i}}^{ 2}$ term might be required for the analysis of *A*_c_, *A*_j_ and *A*_p_ or, in contrast, that steady-state conditions do not hold, particularly, with increased CO_2_ concentrations. Dynamic modelling taking into account the decrease in the values for *g*_m_ or the activity inhibition of key enzymes of the Calvin–Benson–Bassham cycle by CO_2_ acidification could alternatively provide suitable models for the estimation of the biochemical parameters from leaf photosynthesis.

### Electronic supplementary material

Below is the link to the electronic supplementary material.

Supplementary material 1 (PDF 192 kb)

## Figures and Tables

**Fig. 4:**
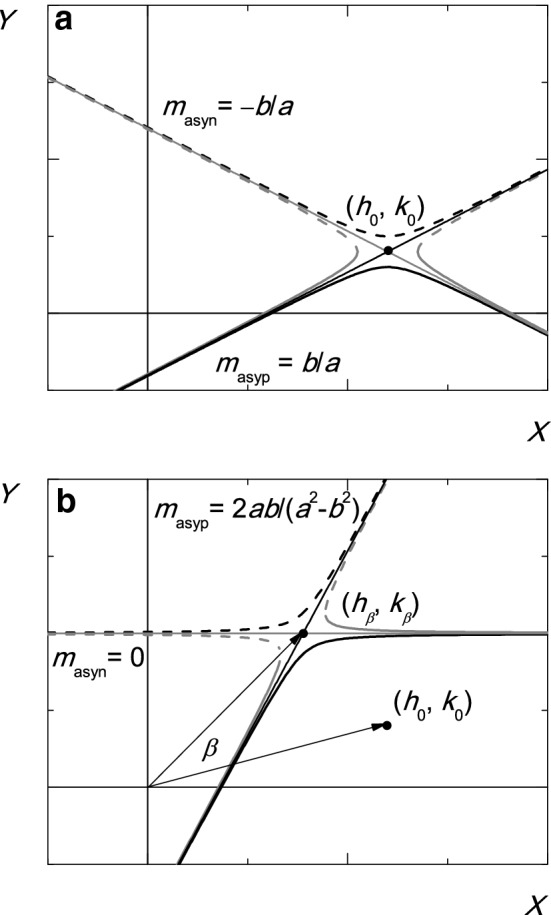
Hyperbolae opening upwards and downwards (thick black lines) and left and right (thick grey lines) in standard form (**a**) and after a rotation of the coordinates by an angle *β* (**b**) together with their positive (thin black line) and negative (thin grey line) asymptotes and their respective centres (black dots). The angle of rotation *β* was chosen to be equal to *b*/*a* and so to fulfil one of the conditions of the quadratic equations of *A*_c_, *A*_j_ and *A*_p_ of the (non)-rectangular hyperbolic FvCB model for C_3_ plants. *A*_c_ and *A*_j_ are rotated hyperbolae opening upwards and downwards, while *A*_p_ opens left and right (see text for further details)

## Appendix 1

The equation of a hyperbola in standard form is as follows:

A1 $$ \frac{{\left( {\dot{x} - h} \right)^{2} }}{{a^{2} }} - \frac{{\left( {\dot{y} - k} \right)^{2} }}{{b^{2} }} = 1,\quad {\text{when}}\;{\text{it}}\;{\text{opens}}\;{\text{left}}\;{\text{and}}\;{\text{right}}\;{\text{or}} $$

A2 $$ \frac{{\left( {\dot{y} - k} \right)^{2} }}{{b^{2} }} - \frac{{\left( {\dot{x} - h} \right)^{2} }}{{a^{2} }} = 1,\quad {\text{when}}\;{\text{it}}\;{\text{opens}}\;{\text{upwards}}\;{\text{and}}\;{\text{downwards}} $$

For each hyperbola, there are two asymptotes with slopes of $\pm {b \mathord{\left/ {\vphantom {b a}} \right. \kern-0pt} a}$ forming an angle *θ* whose value is:

A3 $$ \theta = \arctan \left| {\frac{2ab}{{a^{2} - b^{2} }}} \right| $$

The anticlockwise rotation of the coordinates by an angle β, where

A4 $$ \dot{x} = x\cos \beta + y\sin \beta ,\quad {\text{and}} $$

A5 $$ \dot{y} = - x\sin \beta + y\cos \beta , $$

yields:

A6 $$ \begin{aligned} & y_{\text{LR}}^{2} + y_{\text{LR}} \frac{{\left( {2a^{2} \cos \beta \sin \beta + 2b^{2} \cos \beta \sin \beta } \right)x + 2a^{2} k\cos \beta - 2b^{2} h\sin \beta }}{{b^{2} \sin^{2} \beta - a^{2} \cos^{2} \beta }} \\ & \quad + \,\frac{{\left( {b^{2} \cos^{2} \beta - a^{2} \sin^{2} \beta } \right)x^{2} - (2b^{2} h\cos \beta + 2a^{2} k\sin \beta )x + b^{2} h^{2} - a^{2} k^{2} - a^{2} b^{2} }}{{b^{2} \sin^{2} \beta - a^{2} \cos^{2} \beta }} = 0 \\ \end{aligned} $$

and

A7 $$ \begin{aligned} & y_{\text{UD}}^{2} + y_{\text{UD}} \frac{{\left( {2a^{2} \cos \beta \sin \beta + 2b^{2} \cos \beta \sin \beta } \right)x + 2a^{2} k\cos \beta - 2b^{2} h\sin \beta }}{{b^{2} \sin^{2} \beta - a^{2} \cos^{2} \beta }} \\ & \quad + \,\frac{{\left( {b^{2} \cos^{2} \beta - a^{2} \sin^{2} \beta } \right)x^{2} - (2b^{2} h\cos \beta + 2a^{2} k\sin \beta )x + b^{2} h^{2} - a^{2} k^{2} + a^{2} b^{2} }}{{b^{2} \sin^{2} \beta - a^{2} \cos^{2} \beta }} = 0, \\ \end{aligned} $$

where LR and UD stand for hyperbolae opening left and right, and upwards and downwards, respectively. The only difference between the left-hand sides of Eqs. A6 and A7 is the sign of the term $a^{2} b^{2}$ in the numerator of the last coefficient. The above quadratic equations for non-rectangular hyperbolae opening left and right or upwards and downwards can now be compared, coefficient by coefficient, with each of the quadratic equations of the *A*/*C*_i_ rate curves of the three states c, j and p of the steady-state FvCB model. After the substitution of $C_{\text{c}}$ with $C_{\text{i}} - r_{\text{m}} A$ in Eqs. 2–4, the new *A*_c_, *A*_j_ and *A*_p_ are as follows:

A8 $$ A_{\text{c}}^{2} - A_{\text{c}} \left( {\frac{{C_{\text{i}} + K_{\text{co}} }}{{r_{\text{m}} }} + V_{\text{cmax}} - R_{\text{d}} } \right) + \frac{{\left( {V_{\text{cmax}} - R_{\text{d}} } \right)C_{\text{i}} }}{{r_{\text{m}} }} - \frac{{V_{\text{cmax}} \varGamma^{*} + R_{\text{d}} K_{\text{co}} }}{{r_{\text{m}} }} = 0, $$

A9 $$ A_{\text{j}}^{2} - A_{\text{j}} \left( {\frac{{C_{\text{i}} + 2\varGamma^{*} }}{{r_{\text{m}} }} + {J \mathord{\left/ {\vphantom {J 4}} \right. \kern-0pt} 4} - R_{\text{d}} } \right) + \frac{{\left( {{J \mathord{\left/ {\vphantom {J 4}} \right. \kern-0pt} 4} - R_{\text{d}} } \right)C_{\text{i}} }}{{r_{\text{m}} }} - \frac{{\left( {{J \mathord{\left/ {\vphantom {J 4}} \right. \kern-0pt} 4} + 2R_{\text{d}} } \right)\varGamma^{*} }}{{r_{\text{m}} }} = 0,\quad {\text{and}} $$

A10 $$ A_{\text{p}}^{2} - A_{\text{p}} \left( {\frac{{C_{\text{i}} - \left( {1 + 3\alpha } \right)\varGamma^{*} }}{{r_{\text{m}} }} + 3T_{\text{p}} - R_{\text{d}} } \right) + \frac{{\left( {3T_{\text{p}} - R_{\text{d}} } \right)C_{\text{i}} }}{{r_{\text{m}} }} - \frac{{\left( {3T_{\text{p}} - R_{\text{d}} } \right)\varGamma^{*} }}{{r_{\text{m}} }} = 0 $$

In the first instance, it is observed that the summation of the coefficients of $C_{\text{i}}^{2}$ in each of the three rate curves is zero (Eqs. A8–A10). Therefore, the rotation of the coordinates has to fulfil the condition:

A11 $$ b^{2} \cos^{2} \beta - a^{2} \sin^{2} \beta = 0,\quad {\text{and}}\;{\text{so}} $$

A12 $$ \beta = \pm \arctan \frac{b}{a} + n\pi ,\quad {\text{where}}\;\;n = 0,1,2, \ldots $$

This implies that one of the two asymptotes of *A*_c_, *A*_j_ and *A*_p_ is now parallel to the horizontal axis of the Cartesian coordinate system (i.e. the slope $m_{\text{asy1}} = 0$). Because the rotation does not change the angle between the two asymptotes of the hyperbolae (Eq. A3), *θ* remains constant and so the slope of the second asymptote ($m_{\text{asy2}}$) has to be as follows:

A13 $$ m_{asy2} = {{ \pm 2ab} \mathord{\left/ {\vphantom {{ \pm 2ab} {\left( {a^{2} - b^{2} } \right)}}} \right. \kern-0pt} {\left( {a^{2} - b^{2} } \right)}} $$

The rotation of the coordinates was chosen to be anticlockwise and so the positive value only applies here. The slopes $m_{\text{asy1}}$ and $m_{\text{asy2}}$ will be renamed $m_{\text{asyn}}$ and $m_{\text{asyp}}$, respectively, after the rotation of the coordinates (see below). Figure 4 summarises the main changes in the graphic representation of the two types of hyperbolae and their asymptotes after the rotation of the coordinates.

In the second instance, *A*_c_, *A*_j_ and *A*_p_ share a common term within the second coefficient of the quadratic equations of *A*_c_, *A*_j_ and *A*_p_ (i.e. ${{C_{\text{i}} } \mathord{\left/ {\vphantom {{C_{\text{i}} } {r_{\text{m}} }}} \right. \kern-0pt} {r_{\text{m}} }}$). This implies that for the three equations, the following equality holds:

A14 $$ \frac{{2a^{2} \cos \beta \sin \beta + 2b^{2} \cos \beta \sin \beta }}{{b^{2} \sin^{2} \beta - a^{2} \cos^{2} \beta }} = - \frac{1}{{r_{\text{m}} }} $$

The anticlockwise rotation of the coordinates by $\beta = \arctan {b \mathord{\left/ {\vphantom {b a}} \right. \kern-0pt} a} + n\pi$, where $n = 0$, yields:

A15 $$ \frac{2ab}{{a^{2} - b^{2} }} = \frac{1}{{r_{\text{m}} }} $$

This solution indicates that the oblique asymptotes of three *A*_c_, *A*_j_ and *A*_p_ rate curves have in common their slope, which results equal to the mesophyll conductance to CO_2_ diffusion ($g_{\text{m}} = {1 \mathord{\left/ {\vphantom {1 {r_{\text{m}} }}} \right. \kern-0pt} {r_{\text{m}} }}$).

In the third instance, the second coefficient of each quadratic rate curve is in fact the negative value of the sum of its two asymptotes. If Eqs. A4 and A5 are applied to both the negative and positive asymptotes of the standard forms of the hyperbolae (Eqs. A1 and A2), the following equations are obtained:

A16 $$ y_{\text{asyn}} = \frac{ak + bh}{a\cos \beta + b\sin \beta } + \frac{a\sin \beta - b\cos \beta }{a\cos \beta + b\sin \beta }x = n_{\text{asyn}} + m_{\text{asyn}} x,\quad {\text{and}} $$

A17 $$ y_{\text{asyp}} = \frac{ak - bh}{a\cos \beta - b\sin \beta } + \frac{a\sin \beta + b\cos \beta }{a\cos \beta - b\sin \beta }x = n_{\text{asyp}} + m_{\text{asyp}} x, $$

where $y_{\text{asyn}}$ and $y_{\text{asyp}}$ stand for the new asymptotes after the rotation of the coordinates by *β* (Fig. 4). The sum of the asymptotes is equal to the negative value of the summation of the coefficients of $y_{\text{LR}}$ and $y_{\text{UD}}$ in Eqs. A6 and A7:

A18 $$ y_{\text{asyn}} + y_{\text{asyp}} = - \frac{{\left( {2a^{2} \cos \beta \sin \beta + 2b^{2} \cos \beta \sin \beta } \right)x + 2a^{2} k\cos \beta - 2b^{2} h\sin \beta }}{{b^{2} \sin^{2} \beta - a^{2} \cos^{2} \beta }} $$

If, particularly, $\beta = \arctan {b \mathord{\left/ {\vphantom {b a}} \right. \kern-0pt} a}$, then the slopes have the following values in Eqs. A16 and A17: $m_{\text{asyn}} = 0$ and $m_{\text{asyp}} = {{2ab} \mathord{\left/ {\vphantom {{2ab} {(a^{2} - b^{2} )}}} \right. \kern-0pt} {(a^{2} - b^{2} )}}$. So now we can rewrite the second coefficients of the *A*_c_, *A*_j_ and *A*_p_ rate curves as follows:

A19 $$ y_{\text{asyn}}^{\text{c}} + y_{\text{asyp}}^{\text{c}} = \frac{{C_{\text{i}} + K_{\text{co}} }}{{r_{\text{m}} }} + V_{\text{cmax}} - R_{\text{d}} $$

A20 $$ y_{\text{asyn}}^{\text{j}} + y_{\text{asyp}}^{\text{j}} = \frac{{C_{\text{i}} + 2\varGamma^{*} }}{{r_{\text{m}} }} + {J \mathord{\left/ {\vphantom {J 4}} \right. \kern-0pt} 4} - R_{\text{d}} $$

A21 $$ y_{\text{asyn}}^{\text{p}} + y_{\text{asyp}}^{\text{p}} = \frac{{C_{\text{i}} - \left( {1 + 3\alpha } \right)\varGamma^{*} }}{{r_{\text{m}} }} + 3T_{\text{p}} - R_{\text{d}} , $$

where the superscript indicates the name of the states c, j or p.

In order to know individually the equations for each of the two asymptotes of *A*_c_, *A*_j_ and *A*_p_, one can derive the values of the negative asymptotes ($y_{\text{asyn}}$) from the term that multiplies *x* in the third coefficient of the quadratic equations (Eqs. A6 and A7). This term is, in fact, the negative value of the product between Eqs. A14 and A16, when $m_{\text{asyn}} = 0$. Therefore, Eqs. A19–A21 can now be split as follows:

A22 $$ y_{\text{asyp}}^{\text{c}} = \frac{{C_{\text{i}} + K_{\text{co}} }}{{r_{\text{m}} }}\quad {\text{and}} $$

A23 $$ y_{\text{asyn}}^{\text{c}} = V_{\text{cmax}} - R_{\text{d}} , $$

A24 $$ y_{\text{asyp}}^{\text{j}} = \frac{{C_{\text{i}} + 2\varGamma^{*} }}{{r_{\text{m}} }}\quad {\text{and}} $$

A25 $$ y_{\text{asyn}}^{\text{j}} = {J \mathord{\left/ {\vphantom {J 4}} \right. \kern-0pt} 4} - R_{\text{d}} ,\quad {\text{and}} $$

A26 $$ y_{\text{asyp}}^{\text{p}} = \frac{{C_{\text{i}} - \left( {1 + 3\alpha } \right)\varGamma^{*} }}{{r_{\text{m}} }} \quad {\text{and}} $$

A27 $$ y_{\text{asyn}}^{\text{p}} = 3T_{\text{p}} - R_{\text{d}} . $$

The intersection between the oblique and horizontal asymptotes can be used to know the centre of the hyperbolae. The slopes of the bisecting lines are as follows:

A28 $$ m_{\text{bis}}^{\text{x}} = - r_{\text{m}} - \sqrt {1 + r_{\text{m}}^{2} } ,\quad {\text{where}}\;x\;{\text{stands for}}\;{\text{c}}\;or\;{\text{j}},\;{\text{and}} $$

A29 $$ m_{\text{bis}}^{\text{p}} = - r_{\text{m}} + \sqrt {1 + r_{\text{m}}^{2} } $$

The vertices of each hyperbola can also be derived from the intersections between the equation of the bisecting line—passing through the vertices and the centre of the hyperbola—and the corresponding quadratic equation (Eqs. A8–A10). Table 2 includes a summary of the equations and values that describe the rectangular and non-rectangular hyperbolic equations of the FvCB model.

## Appendix 2

Under steady-state conditions, the net CO_2_ assimilation rate can also be obtained as $A = {{(C_{\text{a}} - C_{\text{i}} )} \mathord{\left/ {\vphantom {{(C_{\text{a}} - C_{\text{i}} )} {r_{\text{s}} }}} \right. \kern-0pt} {r_{\text{s}} }}$, where $r_{\text{s}} = {1 \mathord{\left/ {\vphantom {1 {g_{\text{s}} }}} \right. \kern-0pt} {g_{\text{s}} }}$, if particularly the transpiration rate (*E*) is considered negligible and consequently $g_{\text{s}} \pm {E \mathord{\left/ {\vphantom {E 2}} \right. \kern-0pt} 2} \approx g_{\text{s}}$ (Farquhar and Sharkey 1982). The transition points for the new equations in *A*/*C*_a_ rate curves, when both $r_{\text{m}}$ and $r_{\text{s}}$ are grouped together in the analysis, are now as follows:

For $$ A_{\text{c}} = A_{\text{j}} $$,

A30a, A30b $$ C_{\text{a1}}^{\text{cj}} = C_{\text{c1}}^{\text{cj}} + (r_{\text{s}} + r_{\text{m}} )A_{\text{c1}}^{\text{cj}} \quad {\text{and}}\quad A_{\text{a1}}^{\text{cj}} = A_{\text{c1}}^{\text{cj}} $$

A31a, A31b $$ C_{\text{a2}}^{\text{cj}} = C_{\text{c2}}^{\text{cj}} + (r_{\text{s}} + r_{\text{m}} )A_{\text{c2}}^{\text{cj}}  \quad {\text{and}}\quad A_{\text{a2}}^{\text{cj}} = A_{\text{c2}}^{\text{cj}} $$

for $$ A_{\text{j}} = A_{\text{p}} $$,

A32a, A32b $$ C_{\text{a1}}^{\text{jp}} = C_{\text{c1}}^{\text{jp}} + (r_{\text{s}} + r_{\text{m}} )A_{\text{c1}}^{\text{jp}}  \quad {\text{and}}\quad A_{\text{a1}}^{\text{jp}} = A_{\text{c1}}^{\text{jp}} $$

A33a, A33b $$ C_{\text{a2}}^{\text{jp}} = C_{\text{c2}}^{\text{jp}} + (r_{\text{s}} + r_{\text{m}} )A_{\text{c2}}^{\text{jp}} \quad {\text{and}}\quad A_{\text{a2}}^{\text{jp}} = A_{\text{c2}}^{\text{jp}} $$

and for $$ A_{\text{c}} = A_{\text{p}} $$,

A34a, A34b $$ C_{\text{a1}}^{\text{cp}} = C_{\text{c1}}^{\text{cp}} + (r_{\text{s}} + r_{\text{m}} )A_{\text{c1}}^{\text{cp}} \quad {\text{and}}\quad A_{\text{a1}}^{\text{cp}} = A_{\text{c1}}^{\text{cp}} $$

A35a, A35b $$ C_{\text{a2}}^{\text{cp}} = C_{\text{c2}}^{\text{cp}} + (r_{\text{s}} + r_{\text{m}} )A_{\text{c2}}^{\text{cp}} \quad {\text{and}}\quad A_{\text{a2}}^{\text{cp}} = A_{\text{c2}}^{\text{cp}} $$
