# Supplementary material for: Surfing the Hyperbola Equations of the Steady-State Farquhar–von Caemmerer–Berry C3 Leaf Photosynthesis Model: What Can a Theoretical Analysis of Their Oblique Asymptotes and Transition Points Tell Us?
Source: Bull Math Biol. 2019 Dec 23;82(1):3. doi: 10.1007/s11538-019-00676-z (PMC6952342; doi:10.1007/s11538-019-00676-z)
Supplement: Supplementary file 1 — Supplementary material 1 (PDF 192 kb) [file 11538_2019_676_MOESM1_ESM.pdf]

ELECTRONIC SUPPLEMENTARY MATERIAL

Surfing the hyperbola equations of the steady-state Farquhar-von Caemmerer-Berry C_3 leaf photosynthesis model. What can a theoretical analysis of their oblique asymptotes and transition points tell us?

Bulletin of Mathematical Biology

Jon Miranda-Apodaca, Emilio L. Marcos-Barbero, Rosa Morcuende and Juan B. Arellano

Departamento de Estrés Abiótico. Instituto de Recursos Naturales y Agrobiología de Salamanca, Cordel de merinas, 40-52. 37008 Salamanca, Spain

Corresponding author: Juan B. Arellano. E-mail: juan.arellano@irnasa.csic.es. Phone: +34 923 219 606. Fax: +34 923 219 609

Figure S1

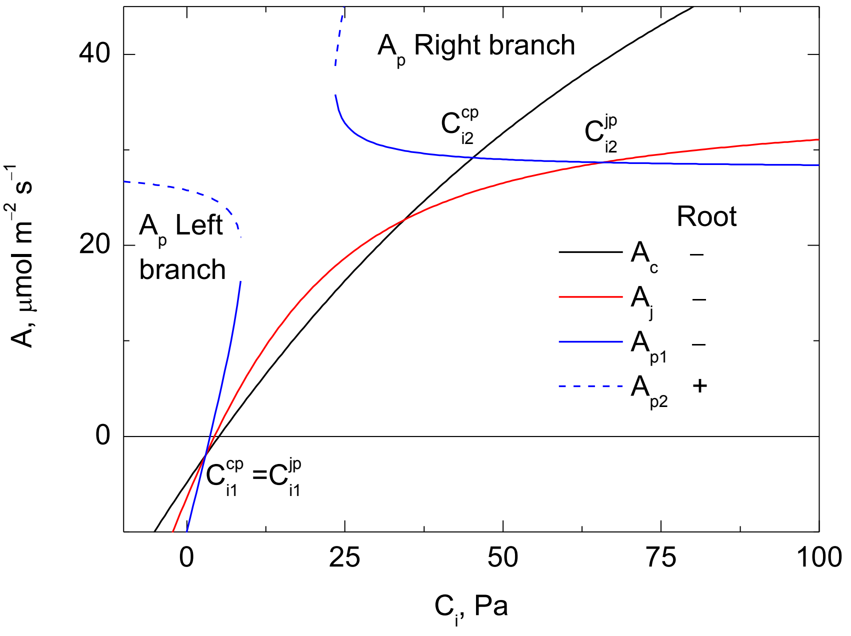

Figure S1. Transition points of A_c and A_j with A_p in the first (C_{i2}^{cp} and C_{i2}^{jp}) and fourth (C_{i1}^{cp} and C_{i1}^{jp}) quadrants of the Cartesian coordinate system in an A/C_i rate curve. In the first quadrant, the transition points are found between the negative roots of the quadratic equations of A_c and A_j and the negative root of the branch opening right of A_p. In the fourth quadrant, the transition points are found between the negative roots of the quadratic equations of A_c and A_j and the negative root of the branch opening left of A_p. The simulation was performed using the following values for the biochemical parameters: V_{cmax}, 100 µmol m^{-2} s^{-1}; J, 150 µmol m^{-2} s^{-1}; T_p, 10 µmol m^{-2} s^{-1}; R_d, 2 µmol m^{-2} s^{-1}; r_m, 0.4 Pa μmol^{-1} m^2 s; K_{co}, 62.1 Pa; Γ^*, 3.74 Pa.
